# Domestication signatures in the non-conventional yeast *Lachancea cidri*

**DOI:** 10.1128/msystems.01058-23

**Published:** 2023-12-12

**Authors:** Pablo Villarreal, Samuel O'Donnell, Nicolas Agier, Felipe Muñoz-Guzman, Jose Benavides-Parra, Kami Urbina, Tomas A. Peña, Mark Solomon, Roberto F. Nespolo, Gilles Fischer, Cristian Varela, Francisco A. Cubillos

**Affiliations:** 1 Departamento de Biología, Facultad de Química y Biología, Universidad de Santiago de Chile, Santiago, Chile; 2 Millennium Institute for Integrative Biology (iBio), Santiago, Chile; 3 Laboratory of Computational and Quantitative Biology, CNRS, Institut de Biologie Paris-Seine, Sorbonne Université, Paris, France; 4 Millenium Nucleus of Patagonian Limit of Life (LiLi), Santiago, Chile; 5 The Australian Wine Research Institute, Glen Osmond, Adelaide, SA, Australia; 6 Instituto de Ciencias Ambientales y Evolutivas, Universidad Austral de Chile, Valdivia, Chile; 7 Center of Applied Ecology and Sustainability (CAPES), Facultad de Ciencias Biológicas, Universidad Católica de Chile, Santiago, Chile; 8 School of Agriculture, Food and Wine, University of Adelaide, Glen Osmond, Adelaide, SA, Australia; University of Massachusetts Amherst, Amherst, Massachusetts, USA

**Keywords:** structural variation, chromosomal rearrangements, adaptation, domestication, *Lachancea cidri*

## Abstract

Evaluating domestication signatures beyond model organisms is essential for a thorough understanding of the genotype-phenotype relationship in wild and human-related environments. Structural variations (SVs) can significantly impact phenotypes playing an important role in the physiological adaptation of species to different niches, including during domestication. A detailed characterization of the fitness consequences of these genomic rearrangements, however, is still limited in non-model systems, largely due to the paucity of direct comparisons between domesticated and wild isolates. Here, we used a combination of sequencing strategies to explore major genomic rearrangements in a *Lachancea cidri* yeast strain isolated from cider (CBS2950) and compared them to those in eight wild isolates from primary forests. Genomic analysis revealed dozens of SVs, including a large reciprocal translocation (~16 kb and 500 kb) present in the cider strain, but absent from all wild strains. Interestingly, the number of SVs was higher relative to single-nucleotide polymorphisms in the cider strain, suggesting a significant role in the strain’s phenotypic variation. The set of SVs identified directly impacts dozens of genes and likely underpins the greater fermentation performance in the *L. cidri* CBS2950. In addition, the large reciprocal translocation affects a proline permease (*PUT4*) regulatory region, resulting in higher *PUT4* transcript levels, which agrees with higher ethanol tolerance, improved cell growth when using proline, and higher amino acid consumption during fermentation. These results suggest that SVs are responsible for the rapid physiological adaptation of yeast to a human-related environment and demonstrate the key contribution of SVs in adaptive fermentative traits in non-model species.

**IMPORTANCE:**

The exploration of domestication signatures associated with human-related environments has predominantly focused on studies conducted on model organisms, such as *Saccharomyces cerevisiae*, overlooking the potential for comparisons across other non-Saccharomyces species. In our research, employing a combination of long- and short-read data, we found domestication signatures in *Lachancea cidri*, a non-model species recently isolated from fermentative environments in cider in France. The significance of our study lies in the identification of large array of major genomic rearrangements in a cider strain compared to wild isolates, which underly several fermentative traits. These domestication signatures result from structural variants, which are likely responsible for the phenotypic differences between strains, providing a rapid path of adaptation to human-related environments.

## INTRODUCTION

Domestication is a co-evolutionary process resulting from a specialized mutualism, in which one species (the domesticator) provides an environment where it actively controls, both the survival and reproduction, of another species (the domesticate) to provide the former with resources and/or services (1). Human-associated domestication, such as for crops and animal farming, is undoubtedly a well-known process in human history (1, 2). Interestingly, while the domestication of some developmentally complex organisms is the result of intentional human behavior, this process happened largely unintentionally in microorganisms (1, 3). The constant interaction of microorganisms in human-related processes allowed their transition from variable and heterogeneous environments, typical of wild ecosystems, to more stable and predictive ones, characteristic of human-related environments, over time (4).

Genomic variation in a population in response to environmental changes is shaped by diverse biological processes, including genetic drift, natural selection, and migration (5, 6). Historically, most studies on genetic variation have focused on single-nucleotide polymorphisms (SNPs), as high-throughput sequencing technologies provide massive amounts of information for comparing species and populations, calibrating divergence times, and inferring adaptive traits of interest (7–12). Recent studies in several organisms, however, have revealed that structural variations (SVs) can sometimes better explain the observed phenotypic diversity of populations (13, 14). SVs are defined as a region of DNA that shows a change in copy number (CNV), orientation, or chromosomal location between individuals (12). SVs can be balanced and have no specific loss or gain of DNA information (inversions and translocations), or they can be unbalanced, where a fraction of the genome is lost or duplicated (insertions, deletions, and duplications) (7, 12). Indeed, experimental evolution assays have indicated that SVs are an important driver of evolution and adaptation to new conditions, such as human-related environments (14).

There are numerous examples in fungal species of physiological adaptations to human-related niches derived from SVs (13, 15–19). For example, wine, beer, and cider represent three human-related environments where different microorganisms are responsible for the fermentation process, including one of the best-studied domesticated microorganisms, the yeast *Saccharomyces cerevisiae*. In wine, greater sulfite resistance in different species across the *Saccharomyces* genus emerged because of major genomic rearrangements, including inversions and chromosomal translocations, impacting the expression of the *SSU1* gene, a sulfite efflux pump (13, 17, 20–22). These studies demonstrated that different genomic variations associated with SVs could be responsible for the rapid adaptation of microorganisms to human-related environments. However, whether there are additional examples of genomic variation events associated with fermentation-related domestication is still largely unknown, particularly in organisms other than *S. cerevisiae*. A better understanding of these events and their consequences can guide biotechnological solutions to improve these industrial processes.

Recent bioprospecting studies in human-related environments, like wine and cheese, have expanded the repertoire of domesticated yeast strains (23). For instance, genomic changes associated with human-related habitats have been reported in different non-conventional yeast genera, such as *Kluyveromyces* and *Torulaspora* (8, 19, 24–26). In all, these studies have deepened our understanding of fungi adaptation to human-related niches; however, additional detailed molecular evidence is needed to understand the adaptative process behind human-made environments and the role of genomic plasticity underlying such adaptation. Cider is a complex fermentative environment where dozens of species interact and sequentially develop throughout the process (27). One of the predominant yeasts in cider fermentation is *Lachancea cidri* (28). This species diverged over 150 MYA from *S. cerevisiae*, it lacks a known sexual cycle, and only haploid strains have been so far recovered (29, 30). This yeast has been isolated from cider fermentation environments in France (reference strain CBS2950), and primary forests in Australia and Patagonia, exhibiting different genetic and phenotypic patterns depending on the isolation environment (30, 31). Interestingly, the CBS2950 and the Australian LC1 *L. cidri* strains are genetically closely related, with only 41 SNPs between their genomes, and a recent estimated divergence between 405-51 years ago, likely associated with human movements (30). However, despite the diverse ecosystems where *L. cidri* strains are present, there is little information about their adaptation to human-related environments at the genetic level.

To study the physiological adaptation of a non-conventional yeast to a human-related environment, specifically, cider fermentation, we explored the genetic landscape of *L. cidri* under different fermentative conditions. We generated telomere-to-telomere genome assemblies of wild strains to identify key genomic signatures underlying phenotypic differences. We focused on SVs and explored how these might have impacted fermentative capacity and could have ultimately allowed a shift from a wild lifestyle to a human-related one. Overall, our study offers new insights into the evolutionary history of a yeast species and explains how human-related environments can prompt genome reorganization as a signature of domestication.

## RESULTS

### Human-related *Lachancea cidri* strain exhibits phenotypic differences under fermentative conditions compared to wild strains

To explore phenotypic differences between wild and putatively domesticated cider *L. cidri* strains, we measured the fermentation kinetics (in terms of CO_2_ production) and the production of fermentation-derived metabolites in two natural musts: apple juice, to produce cider, and Chardonnay grape must, to produce wine. In this instance, we selected Chardonnay as it closely mirrors a fermentation must similar to cider, characterized by comparable sugar concentrations, acidity, and potential for a wide range of flavors (32). For both assays, we considered three strains previously obtained and characterized at the genomic and phenotypic levels (see reference (30)) considering the following criteria: (i) the human-related strain *L. cidri* CBS2950, obtained from a cider fermentative environment in France, (ii) the wild LC1 strain, which is the closest known relative to CBS2950 and was isolated from tree sap samples in the Central Plateau of Tasmania, Australia, and (iii) NS18, a wild *L. cidri* strain from *Nothofagus* forests from Patagonia (Table S1) (30, 33). The commercial strain *S. cerevisiae* Lalvin EC1118 was used as a control.

First, we looked at the ability of the strains to produce CO_2_ (g/L) and other metabolites during the fermentation. No significant differences in fermentation kinetics were observed among the *L. cidri* strains during cider fermentation. The three *L. cidri* strains showed a similar CO_2_ loss (g/L) profile (*P*-value > 0.05, one-way ANOVA) compared to the *S. cerevisiae* Lalvin EC1118 commercial control strain (*P*-value > 0.05, one-way ANOVA), demonstrating their remarkable ability to ferment this apple must (Fig. S1A). Furthermore, while no differences were observed among the *L. cidri* strains in the production of various metabolites, such as ethanol, glycerol, and succinic acid (Fig. S1, p*-value* > 0.05, one-way ANOVA), in the case of ethanol and succinic acid their production was higher in these strains than in the control (Fig. S1B-D *P-value* < 0.05, one-way ANOVA).

**Fig 1:**
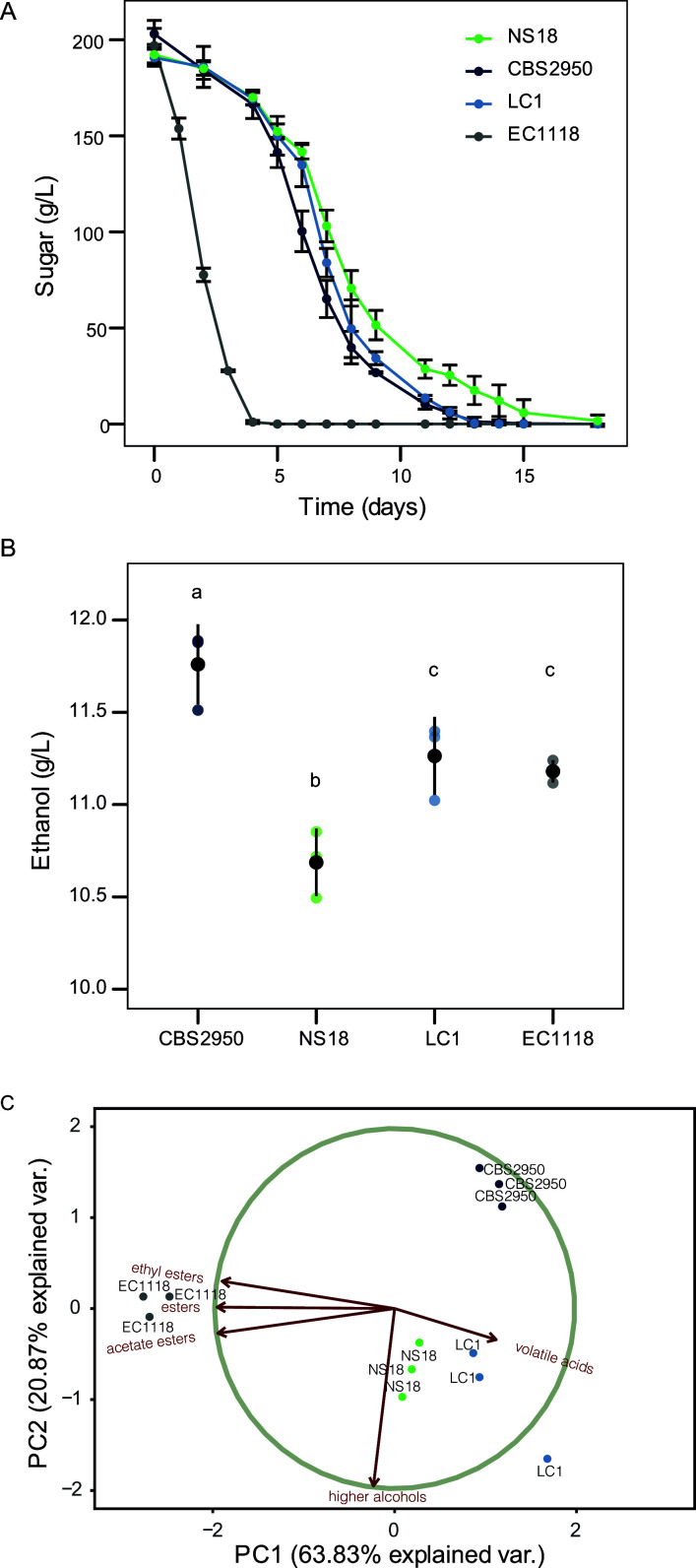
*L. cidri* fermentation performance under Chardonnay wine must. (**A**) Kinetics of sugar consumption (g/L) in Chardonnay fermentations for three *L. cidri* strains (CBS2050/Cider; LC1/Australia, and NS18/Chile). Experiments were carried out in triplicates. (**B**) Ethanol production (g/L) at the end of the Chardonnay fermentation. (**C**) Principal component analysis of volatile compound production in Chardonnay fermentation. Each dot represents an independent measurement. Different letters reflect statistical differences between strains with a *P*-value < 0.05, one-way analysis of variance (ANOVA).

We selected the Chardonnay grape due to its similarity in sugar composition with the apple juice (cider). In this case, the three *L. cidri* strains completed the fermentation after 15 days, while the *S. cerevisiae* control strain finished in 4 days (Fig. 1A). Yet, at the end of the Chardonnay fermentation, ethanol concentration was significantly higher in the cider strain CBS2950 compared to that in all of the other strains, including the *S. cerevisiae* control (*P*-value < 0.05, one-way ANOVA) (Fig. 1B). While no significant differences among the *L. cidri* strains were observed to produce glycerol and succinic, citric, lactic, and acetic acids (Fig. S2, *P-value* > 0.05, one-way ANOVA), the *S. cerevisiae* control strain produced higher succinic and citric acid concentrations than all *L. cidri* strains (Fig. S2, *P-value* > 0.05, one-way ANOVA). A particular case was the production of malic acid, where the lowest production was observed in strain NS18 (Fig. S2, p*-value* < 0.05, one-way ANOVA), while CBS2950 and LC1 did not show significant differences between them (Fig. S2, *P-value* > 0.05, one-way ANOVA).

Distinctive production profiles of volatile compounds (VCs) were observed for strains isolated from the fermentative environments, *L. cidri* CBS2950 and *S. cerevisiae* Lalvin EC1118, compared to wild strains (Table S2). To reduce the dimensionality of the data set and interpret the VC production for each strain, a global principal component analysis (PCA) using the VCs data was performed (Fig. 1C). The first two components explained 63.8% and 20.9% of the observed variation, respectively, separating the strains according to their isolation source (forests, wine, or cider) (Fig. 1C). The CBS2950 strain produced lower concentrations of higher alcohols and acetate esters (*P*-value < 0.05, one-way ANOVA, Table S2) than the other *L. cidri* strains, demonstrating a distinctive organoleptic profile and revealing a clear phenotypic differentiation from the wild strains.

Altogether, these results highlight phenotypic differences between the CBS2950 strain compared to wild *L. cidri* strains. In particular, the CBS2959 strain shows the best profile in terms of ethanol production levels and a different VC profile, suggesting the presence of tolerance mechanisms to ethanol during fermentation, and differences in the consumption of VCs precursors.

### *Lachancea cidri* isolates exhibit different fermentation capacities under varying nitrogen concentrations

**Fig 2:**
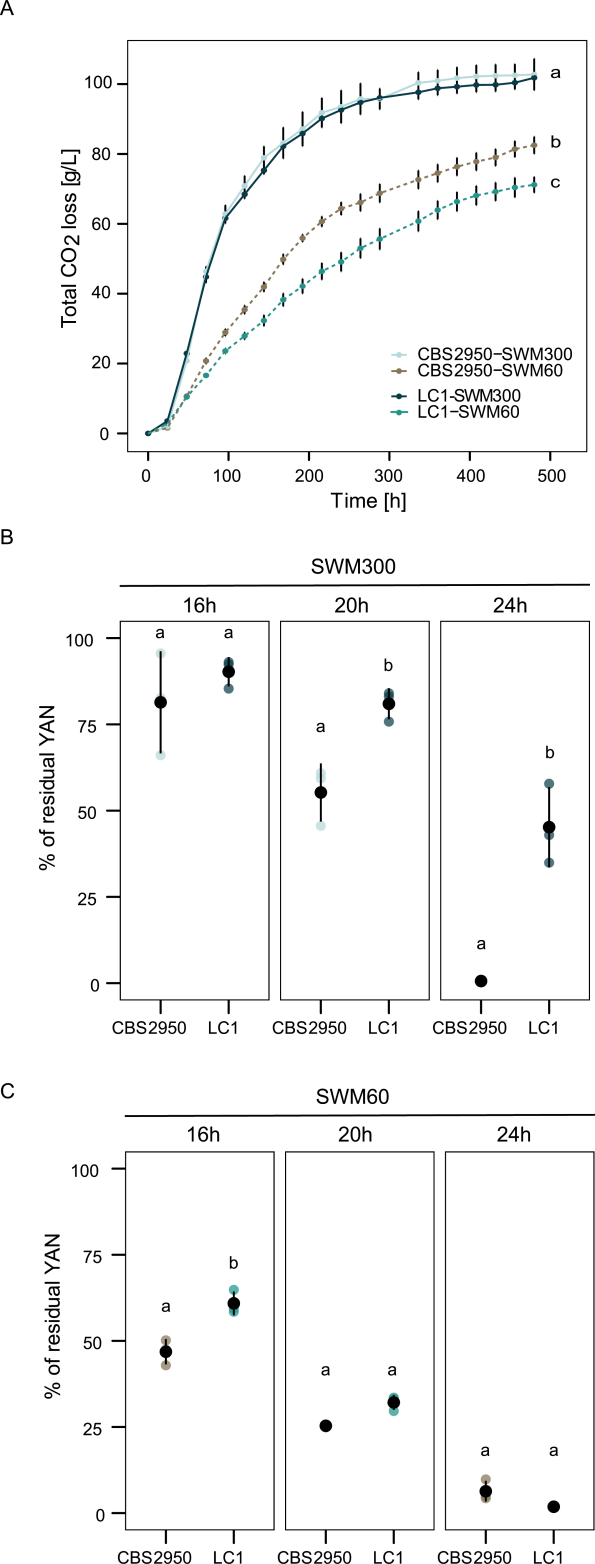
*Lachancea cidri* fermentation performance under synthetic wine must. (**A**) Fermentation kinetics in Synthetic Wine Must (SWM). The solid line shows the fermentation profiles of the CBS2950 and LC1 strains at high nitrogen concentrations SWM300 (300 mg/mL YAN), and the dashed line at low nitrogen concentrations (60 mg/mL YAN) (**B**) Percentage of residual nitrogen at different time points in SWM300 fermentation (measured at 16, 20, and 24 hours). (**C**) Percentage of residual nitrogen at different time points in SWM60 fermentation. Different letters reflect statistical differences between strains with a *P*-value < 0.05, one-way analysis of variance (ANOVA).

To evaluate whether phenotypic differences between wild and cider *L. cidri* strains are related to nitrogen consumption, we performed micro-fermentation assays under different nitrogen conditions, comparing CBS2950 and the LC1 strain. For this, micro-fermentations using Synthetic Wine Musts (SWM) with different yeast assimilable nitrogen (YAN) concentrations, SWM300 (300 mg/mL YAN) and SWM60 (60 mg/mL YAN), were performed. In SWM300, no differences were observed at the end of the fermentation between these *L. cidri* strains regarding fermentation kinetics or total CO_2_ production (*P*-value > 0.05, one-way ANOVA) (Fig. 2A). Conversely, a decrease in the amount of YAN (SWM60) had a significant impact on the fermentation performance, and the extent of the effect depended on the strain (*P*-value < 0.05, one-way ANOVA, Fig. 2A). Under low-nitrogen conditions, the CBS2950 cider strain exhibited a higher fermentative capacity, suggesting a greater ability to ferment under low-nitrogen conditions (Fig. 2A). The CBS2950 strain exhibited a higher and more efficient nitrogen utilization profile than the wild strain LC1 early in the fermentation under both conditions (*P*-value < 0.05, one-way ANOVA, Fig. 2B and C; Table S3). However, much more efficient nitrogen consumption in SWM300 did not necessarily result in greater fermentation capacity (Fig. 2B). This contrasts with the situation in SWM60, where faster nitrogen consumption at earlier time points translated into higher CO_2_ loss levels in the cider strain (Fig. 2C).

To evaluate whether fermentation differences impacted the secondary metabolite production, we estimated ethanol, glycerol, and organic acids production. No differences were observed in the production of secondary metabolites (ethanol, glycerol, acetic acid, and succinic acid) between the CBS2950 and LC1 in SWM300 (*P*-value > 0.05, one-way ANOVA) (Fig. S3) However, in SWM60, differences between CBS2950 and LC1 were observed in the production of glycerol, acetic acid, and succinic acid (*P*-value < 0.05, one-way ANOVA) (Fig. S3 C and D). Overall, these results highlight differences in the fermentation profiles depending on the available nitrogen conditions in synthetic wine must.

### The human-related *Lachancea cidri* strain features major chromosomal rearrangements compared to wild strains

**Fig 3:**
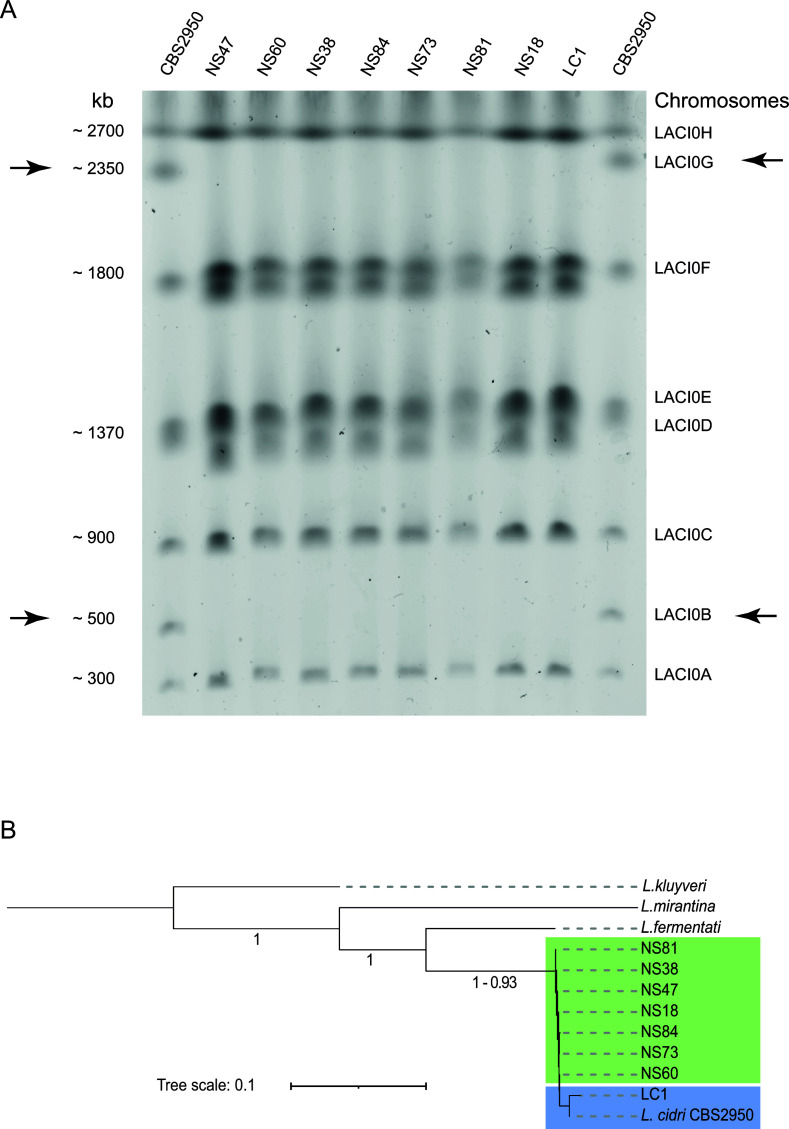
Molecular karyotype and phylogeny of *L. cidri* strains. (**A**) Pulsed-field electrophoresis of the chromosomal DNA of nine *L. cidri* strains. *L. cidri* CBS2950 is located in the first and last column. Wild South American strains: NS47, NS60, NS38, NS84, NS73, NS81, and NS18. Wild Australian strain: LC1. Black arrows show the chromosomes with a different electrophoretic pattern between wild and cider strains. (**B**) Consensus phylogenetic tree of *L. cidri* long-read genomes. The tree was built by orthogroup inference. Branch lengths represent the average number of substitutions per site across the sampled gene families. *L. mirantina*, *L. kluyveri,* and *L. fermentati* were used as outgroups.

Given the significant phenotypic differences among *L. cidri* strains, our objective was to elucidate the genetic underpinnings responsible for these variations. Previously, we conducted whole-genome sequencing of the CBS2950 and LC1 *L. cidri* strains. Surprisingly, the comparison revealed a minimal genomic disparity, consisting of only 41 single nucleotide polymorphisms (SNPs) scattered across the genome. Notably, none of these SNPs appeared to affect protein-coding sequences (30). To identify the genetic basis of the phenotypic differences between these two strains, we decided to explore genetic changes beyond SNPs and to evaluate the presence of SVs. For this, we initially performed a karyotyping analysis, followed by long-read sequencing. The molecular karyotype using CBS2950 and LC1 strains, plus seven wild strains from different Patagonian localities, was first performed to identify major rearrangements in the genome, using pulse-field electrophoresis (Fig. 3A; Table S1). The chromosomal DNA of the different strains was separated into eight electrophoretic bands ranging in size from 300 to 2,700 kb (Fig. 3A). Chromosomes LACI0B and LACI0G exhibited a unique migration pattern in CBS2950, with significant size variation compared to the other strains (Fig. 2A).

Whole-genome long-read Nanopore sequencing was used to assemble the genome of the eight wild *L. cidri* strains (one contig per chromosome) in an attempt to identify the origin of the chromosome size differences (Fig. S4, Table S4, and S5). Using a consensus species tree constructed through orthogroup inference with 3,408 orthologs, we observed clustering between CBS2950 and LC1 (Fig. 3B), confirming a high level of genetic relatedness between these two strains. This finding aligns with our previous studies (30). Strains isolated from wild environments are at the base of the species tree, while *L. cidri* CBS2950 recently diverged from the Australian clade.

**Fig 4:**
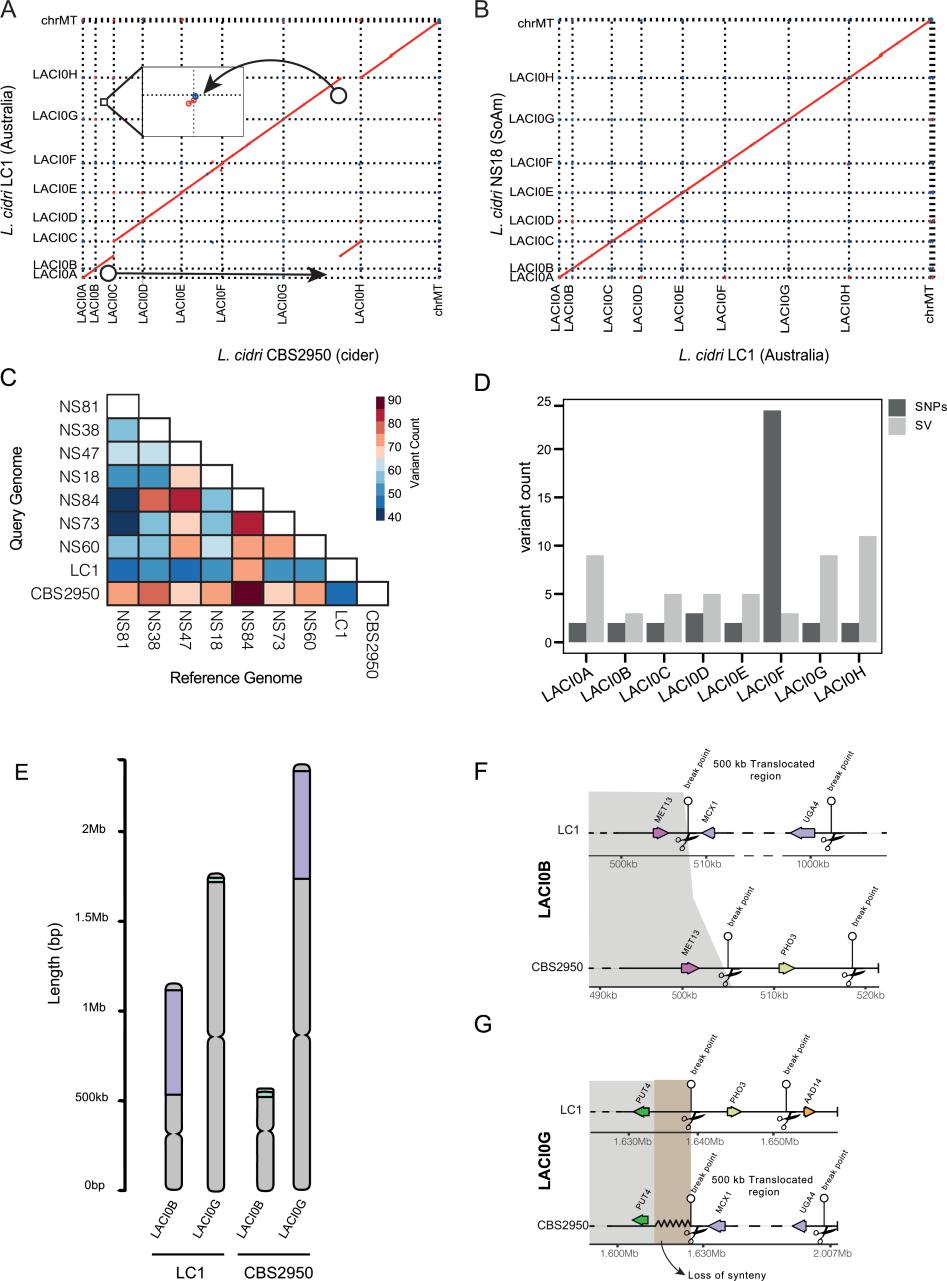
*L. cidri* long-read genome assembly. (**A**) Genome synteny analysis of the reference strain CBS2950, and the Australian LC1 strain. The dot plot representation depicts the DNA sequence identity between the two *L. cidri* genomes, where a reciprocal translocation is apparent between chromosomes B and H. (**B**) Genome synteny analysis of two wild *L. cidri* strains LC1 and NS18. Dot plot representation of DNA sequence identity. Red dots indicate forward matches and blue dots for reverse matches. (**C**) SVs pairwise comparisons among all *L. cidri* genome assemblies with the total number of structural variations. (**D**) LACI0B and LACI0G chromosome comparison between wild and CBS2950 strains. Purple and green colors denote translocated regions. (**E**) Number of SVs and SNPs per chromosome between CBS2950 and LC1 (**F**) LACIB Chromosome synteny (**G**) LACIG Chromosome synteny. Black scissors show breakpoints in the genome.

We then evaluated the presence of SVs in these strains by comparing all *de novo* assemblies against the *L. cidri* CBS2950 strain. Perfect collinearity was observed in all chromosomes, except for chromosomes LACI0B and LACI0G in the CBS2950 strain (Fig. 4A). This strain showed a ~16 Kb and 500 Kb terminal translocation between chromosomes LACI0B and LACI0G, respectively (Fig. 4A), which is consistent with the karyotyping results (Fig. 3A). Comparing two wild strains randomly, perfect collinearity was observed across all chromosomes, demonstrating that chromosome size differences are specific to the cider strain (Fig. 4B). To quantify the extent of all SVs across the genomes, a comprehensive analysis using pairwise comparisons (MUM&Co) was performed (Fig. 4C). Six types of SVs were evaluated: deletions, insertions, duplications, contractions, inversions, and translocations (Table S6). We found that the cider strain had a higher amount of total variation (mean = 71.1 SVs count between CBS2950 and the other wild strains) compared to the closely related wild strain *L. cidri* LC1 (mean = 52.8 SVs count between LC1 and the other eight sequenced strains) (Fig. S5A), demonstrating enrichment of SVs relative to SNPs in CBS2950 across the genome (Fig. 4D). Most of the structural variants were localized in sub-telomeric regions (Fig. S5B), mainly corresponding to unbalanced variants (Fig. S6C). Overall, the CBS2950 strain showed a higher number and larger size of SVs than the other wild strains (Fig. S6A and B), with the most frequent SV being deletions (Fig. S7A). In addition, we found in CBS2950 a higher frequency of SVs impacting intergenic regions compared to coding sequences (Fig. S7B), which may affect gene expression.

To compare the impact of SV on gene order, we performed a gene order synteny comparison across the genome between the CBS2950 and LC1 strains. A perfect synteny was observed between the CBS2950 strain and LC1, except for the LACI0GtLACI0B translocation (Fig. 4A and E). This translocation comprises 226 genes, which are relocated from chromosome LACI0B to chromosome LACI0G in the CBS2950 strain. The translocation breakpoint also impacted the regulatory region of the *PUT4* gene in the cider strain, which encodes for a proline permease required for high-affinity proline transport. These genomic data demonstrate a large genetic remodeling in the domesticated *L. cidri* CBS2950 strain compared to its wild counterpart.

### Differential gene expression in the human-related *Lachancea cidri* strain

**Fig 5:**
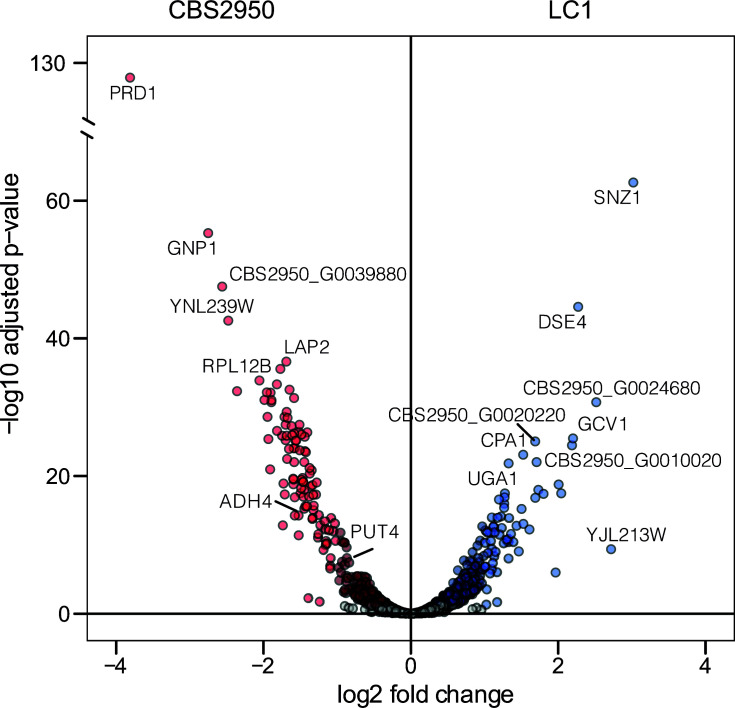
Differential gene expression between wild and cider *L. cidri* strains under synthetic wine fermentation conditions. A. The volcano plot depicts differentially expressed genes (1-fold change in expression; *P*-adjusted <0.05). Up-regulated and down-regulated genes in CBS2950 relative to LC1 are depicted in red and blue dots, respectively.

Having identified a significant genomic rearrangement between the CBS2950 cider and LC1 strains, we proceeded to evaluate whether this extensive chromosomal alteration could impact the expression of multiple genes and potentially contribute to the observed phenotypic differences between the strains, particularly under low nitrogen conditions. We chose to investigate the strains’ expression profiles under low nitrogen conditions due to our previous results of substantial phenotypic disparities between them in this specific environment. Subsequently, we performed RNA-seq under low-nitrogen wine fermentation conditions (SWM60). Differentially expressed gene (DEG) analysis revealed 569 DEGs across the whole genome between the strains (either up- or down-regulated, FDR < 0.05, Table S7). Interestingly, several fermentation and nitrogen-related genes were found as DEGs. These included *GNP1* (broad specificity amino acid permease) and *LAP2* (cysteine aminopeptidase with homocysteine-thiolactone activity), both related to amino acid transport. Both genes were up-regulated in the cider strain relative to LC1 (Fig. 5A). *ADH4* (alcohol dehydrogenase isoenzyme IV) was also up-regulated in CBS2950 (Fig. 5A), consistent with its greater fermentative capacity. Enrichment analysis of gene ontology (GO) terms associated with DEGs highlighted the up-regulation of ribosomal genes in CBS2950, and the down-regulation of secondary metabolite production like organic acids and carboxylic acid (Table S8). Interestingly, these genes were up-regulated in the wild strain (Table S9), which could explain the differences in the VCs profiles found in Chardonnay fermentation, where the wild strain produces a greater repertoire of aromas compared to the cider strain.

Interestingly, expression differences were not enriched in the translocated genes within the LACI0B and LACI0G chromosomes (*P*-value > 0.05 Hypergeometric Test, Total genome *P*: 0.142; Translocated region *P*: 0.150), indicating that the translocation did not significantly affect the expression of these genes under these conditions. We also analyzed the region surrounding the LACI0GtLACI0B translocation breakpoints, in the vicinity of the *PUT4* and *PHO3* promoter regions (Fig. 3G). While no differences were found for *PHO3, PUT4* was differentially expressed between CBS2950 and LC1 (*P*-adjusted <0.05, Table S7). In this case, the cider strain exhibited greater *PUT4* expression levels compared to those in LC1, suggesting an effect of the SV on the gene’s expression profile (Table S7). Altogether, these results suggest that changes in the expression of various genes related to nitrogen metabolism may underlie some of the observed phenotypic differences between CSB2950 and LC1 strain under fermentative conditions.

### Human-related *Lachancea cidri* strain exhibits a series of phenotypic domestication signatures

**Fig 6:**
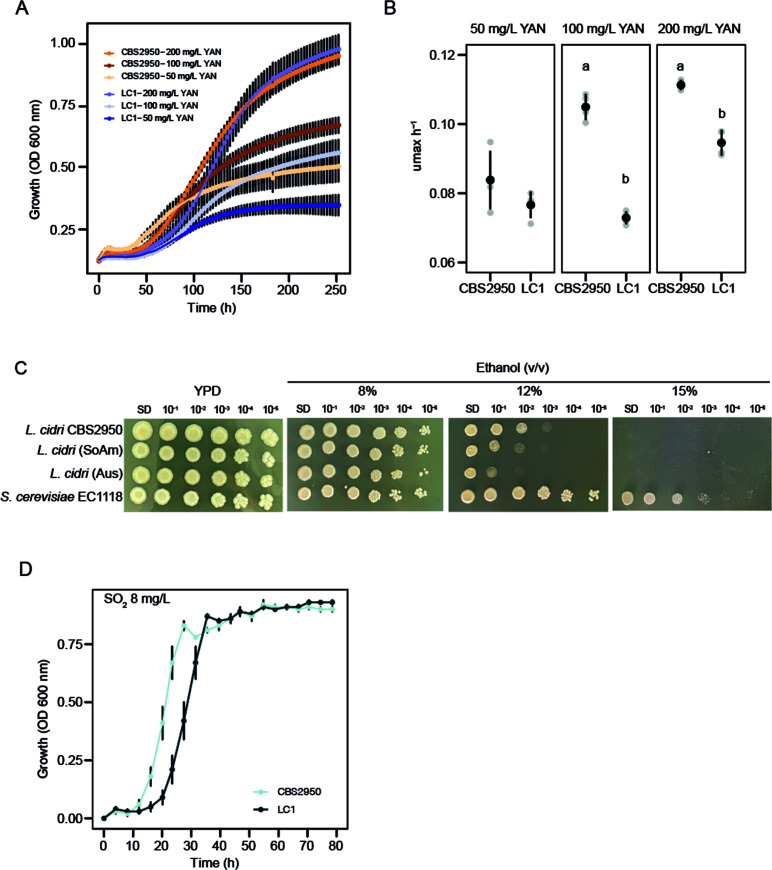
Domestication signatures in *Lachancea cidri*. A. Growth curve at different proline concentrations (mg/L YAN) in the cider (CBS2950) and wild *L. cidri* strains. B. Growth rate at different concentrations of proline (mg/L YAN). Different letters depict statistical differences between strains with a *P*-value < 0.05, one-way analysis of variance (ANOVA). C. Comparison of ethanol tolerance between the CBS2950 strain and wild (SoAm = NS18, Aus = LC1) strains of *L. cidri. S. cerevisiae* EC1118 was used as a control. D. Effect of sulfite (SO_2_ 8 mg/L) on the growth of *L. cidri* strains.

To determine the presence of domestication signatures in the cider strain, different phenotypic assays representative of signatures in the fermentation process were performed. Given the effect of the translocation event on the expression of *PUT4*, we first evaluated microbial growth using proline as the sole nitrogen source (Fig. 6A). At each proline concentration assessed, a greater maximum optical density (Fig. 6A) and growth rate (μmax) (Fig. 6B) were observed in CBS2950 compared to LC1 (*P*-value < 0.05, one-way ANOVA). To determine whether this effect is specific to proline, we used a combination of four different amino acids (glutamic acid, aspartic acid, alanine, and leucine) at 50 and 200 mg/L YAN, and a complete YNB 2% glucose media (Fig. S8). In all cases, no significant differences were observed between the two strains (*P*-value > 0.05, one-way ANOVA, Fig. S8), suggesting that the growth differences observed are specific to proline.

Considering the correlation between the reported function of the *PUT4* gene and ethanol tolerance (34), we also evaluated colony growth under various ethanol concentrations in all *L. cidri* strains (Fig. 6C). A similar growth was observed for all strains at 8% vol/vol, whereas at higher ethanol concentrations (12% vol/vol) CBS2950 showed improved growth performance, suggesting a greater tolerance and adaptation to high-ethanol concentrations, such as those found in cider fermentation (Fig. 6C). In this way, the expression differences in *PUT4* correlated with greater microbial growth when proline was the sole nitrogen source and may be responsible for increased ethanol tolerance in the “domesticated” *L. cidri* CBS2950 strain compared to the wild *L. cidri* LC1 strain. These results suggest a major role of the SV breakpoint in CBS2950 adaptation to cider environments.

Microbial growth under sulfite (SO_2_) conditions, which is another signature of adaptation to human-related fermentative environments, was also evaluated (Fig. 6D and Fig. S9). CBS2950 exhibited greater growth performance compared to LC1 under the different SO_2_ concentrations tested (Fig. 6D), exhibiting a higher growth rate and a shorter lag phase compared to the native LC1 strain (Fig. S9). Given this, we examined the presence of SVs in *SSU1*, a gene that encodes for a sulfite efflux pump known to confer sulfite (SO_2_) resistance (13). Mum&Co and manual analysis of the *SSU1* neighborhood, however, revealed no differences between CBS2950 and LC1.

Altogether, these results demonstrate the presence of different phenotypic advantages under fermentative conditions in the *L. cidri* CBS2950 strain, and these can partly be explained by balanced SVs. This highlights the potential importance of these genomic rearrangements in providing an adaptive advantage to conditions found in human-related fermentative environments.

## DISCUSSION

Domestication is a major selective force characterized by several genomic signatures, such as increased CNV, differential gene expression, and major genomic rearrangements (SVs) that impact chromosomal organization (13, 24, 26, 35). Different forms of genomic reorganization allowing microorganisms to rapidly adapt to novel environmental challenges have been well documented in various model organisms, including yeast and fungi of the *Saccharomyces* and *Penicillium* genera (4, 10, 13, 20, 35–37). *Saccharomyces* yeast species have anthropological significance with industrial and biotechnological applications and have been established as suitable model systems for evolutionary, genetics, and medical studies (38–40). The genetic and phenotypic diversity in other yeast genera, and the many relevant key insights that could be obtained from studying them, however, remain largely overlooked (3), particularly for studying adaptation.

Here, we looked outside the *Saccharomyces* genus to understand additional mechanisms of adaptation/domestication related to human-related environments in other yeast species. We assessed domestication signatures in *Lachancea cidri*, a haploid species with an unknown sexual cycle, recently isolated from a cider fermentative environment in France (strain CBS2950), and from tree samples in Australia and Patagonia (29, 30). Our fermentation analyses demonstrate specific traits in the domesticated *L. cidri* CBS2950 strain that appear to provide a fitness advantage under fermentative conditions compared to wild strains. Our results are in agreement with previous reports where yeasts adapted to fermentative conditions showed an increased ethanol tolerance and more efficient amino acid consumption (3, 13, 27, 34, 41–48). Interestingly, we found a differential volatile compounds profile in wine fermentation by CBS2950. The CBS2950-derived wine has a less complex profile than that from native strains, a feature previously reported as a consequence of human selection (1, 4, 10, 39, 43, 49–51).

Using long- and short-read sequencing strategies, we demonstrated an enrichment of SVs in the CBS2950 strain compared to wild strains, which may partly be responsible for some of the phenotypic differences observed. Reports on other non-conventional yeasts have highlighted the presence of SVs with major impacts on the adaptation to new environments (8, 19, 24, 25), representing a rapid and efficient strategy to promote phenotypic changes to artificial environments. Indeed, several genomic modifications that impact the ability to ferment lactose and, thus, to adapt efficiently to dairy farmers’ environments were recently identified in *Kluyveromyces lactis* var. *lactis* (25). Moreover, *K. lactis* gained the ability to metabolize lactose from a horizontal gene transfer event from *Kluyveromyces marxianus* (19), emerging as a correlated response of the adaptation to the dairy processes (19, 25). Similarly, the acquisition of a cluster of *GAL* genes and the expansion and functional diversification of *MAL* genes were reported as domestication signatures to dairy and bread products in *Torulaspora delbrueckii* (8). Similar observations have been made in plants and fish, such as the Asian rice (*Oryza sativa*) and lake whitefish (*Coregonus* sp.), SVs contribute substantially to reshaping the genome architecture, underlying speciation events, and species differentiation with unique traits to adapt to human-related environments (52, 53).

For *L. cidri*, its haploid condition might promote major genomic rearrangements to generate a cost-effective adaptive change. Although single-nucleotide polymorphisms were initially hypothesized to underlie most selectable variation (12), we demonstrate here that genetic rearrangements like SVs might represent a major source of genetic variation under fermentative conditions in this species. Chromosomal rearrangements can underlie adaptation by affecting the expression of genes located in the proximity of the SVs breakpoints *via* gain, loss, or movement of regulatory regions (54). Here, we report the effect of a large translocation on the *PUT4* gene, located in the SV breakpoint neighborhood. RNA-seq analyses showed higher expression levels of *PUT4* in the cider strain, suggesting that the SV shed light on the molecular origin of the phenotypic differences between strains. Interestingly, these higher expression levels agree with increased ethanol tolerance in the “domesticated” cider strain. In this sense, the alcohol content in ciders can varies between 1.2% and 8.5% vol/vol (55). Consistent with this, previous studies have shown that an increase in proline consumption greatly improves tolerance to high ethanol concentrations (34). Thus, the SV breakpoint may have played a role in improving ethanol tolerance, amino acid consumption, and microbial growth in CBS2950. SVs can influence gene expression and impair gene function, which may result in signatures of local adaptation (9). Although our assay primarily focused on wine rather than cider, we conducted our experiments using a synthetic wine must as the experimental medium. This approach allowed us to modulate nitrogen concentrations, an advantage not easily achievable in cider, where control over this variable is limited. In apples, the initial nitrogen content ranges between 27 and 574 mg/L and is directly related to the amino acid content, with aspartic acid, glutamic acid, asparagine, serine, and proline being the main sources (56). Consequently, we believe that under low nitrogen conditions in cider, *L. cidri* strains may exhibit a fitness advantage over other species.

Another common domestication signature that has been associated with SVs is SO_2_ tolerance (13, 24). SO_2_ is widely used in cider fermentation as an antioxidant and an inhibitor of oxidizing enzymes (57). While we did observe increased tolerance to SO_2_ in CBS2950 compared to wild strains, we did not detect an SV associated with the *SSU1* gene or in its regulatory region. The *SSU1* gene is located approximately 540 kb upstream of the LACI0G translocation, suggesting an unknown molecular mechanism by which the “domesticated” strain performs better under high SO_2_ concentrations. This mechanism may differ from that found in other domesticated *Saccharomyces* strains, where the increase in tolerance has been directly attributed to differential expression of the *SSU1* gene due to modifications in the regulatory region of the gene (13, 24). However, it is important to acknowledge that our study is constrained by the utilization of a single *L. cidri* strain isolated from cider, limiting the scope of our results. Considering the current limitations in strain availability, specifically the scarcity of publicly accessible collections featuring *L. cidri* strains from Europe and cider sources, our investigation has primarily focused on the strains currently available in public repositories. These include the CBS2950 strain, isolated from cider as of the current date, and supplemented with wild strains from Australia and Chile. In the future, broader bioprospecting efforts may shed light on the prevalence of these SVs and domestication signatures across *L. cidri* strains obtained from human-related environments.

In conclusion, our study reveals that SVs may account for most of the genetic variation between cider and wild strains in *L. cidri*. Despite SVs being infrequent in natural populations (54), we observed a high abundance of these SVs between the CBS2950 and LC1 strains, greater than the frequency of single nucleotide polymorphisms (SNPs). These SVs could play a vital role in enabling organisms to adapt to human-related environments, such as cider. In particular, such events may have resulted in the ability of *L. cidri* to efficiently consume amino acids and tolerate high ethanol concentrations, allowing it to thrive in a fermentative environment. The different “domestication signatures” we observed for *L. cidri* under fermentation are similar to those found in *S. cerevisiae* strains under similar conditions (ethanol tolerance, efficient consumption of amino acids, and SO_2_ tolerance), suggesting convergent adaptive changes to human-related environments in yeasts, despite over 100 MYA of divergence. These findings provide additional insights into microbial domestication and broaden the perspective of the fitness effects of SVs in yeast species associated with human-related environments.

## MATERIALS AND METHODS

### Yeast strains

Nine *L. cidri* strains, previously obtained from cider in France, bark samples in Chilean Patagonia and Australian forests, are representative of the different lineages in the species. These strains were selected for long-read sequencing and genome assembly (Table S1, (29, 30)). The genome assembly and annotation of the *L. cidri* CBS2950 strain were obtained from the GRYC (Genome Resources for Yeast Chromosomes, INRA, France).

### Fermentation performance in wine and cider

Fermentation assays were performed under three different must conditions: Cider, Chardonnay, and synthetic wine must (SWM). Cider fermentation was performed in natural green apple juice (Afe). Chardonnay grape juice was obtained from Blewitt Springs, Clarendon Hills (South Australia). Chardonnay juice contained 195 g/L of sugar (equal amounts of glucose and fructose), 8 mg/L of free sulfur dioxide, and had a pH of 3.31. Yeast assimilable nitrogen (YAN) was adjusted to 250 mg N/L by adding diammonium phosphate (DAP). SWM was prepared at 300 mg/mL YAN (SWM300) and 60 mg/mL YAN (SWM60) as previously described (58).

For each fermentation, the strains were initially grown under constant agitation in 10 mL of YPD medium (1% yeast extract, 2% peptone, and 2% glucose) for 24 hours at 20°C. 1 × 10^6^ cells/mL were inoculated into 50 mL of each must (in 200 mL flasks) and incubated at 25°C with constant agitation. Cider and SWM fermentations were weighed every day to calculate the accumulated CO2 loss. Chardonnay fermentation samples were taken regularly to monitor fermentation by measuring sugar concentration in culture supernatants using high-performance liquid chromatography (HPLC). At the end of the fermentation, samples were centrifuged at 9,000× g for 10 min, and the supernatant was collected for extracellular metabolite determination as previously described (58). Volatile compounds were measured as previously described (18, 49).

### DNA extraction and long-read sequencing

DNA was extracted from native *L. cidri* strains as previously described in reference (59). Libraries were sequenced on an R9.4 flow cell using a Minion (Oxford Nanopore Technologies, UK). The raw fast5 files were transformed to fastq files and debarcoded using Guppy v5.0.14 with the “super high accuracy” model (https://nanoporetech.com/accuracy). Barcodes and adapters sequences were trimmed using Porechop (https://github.com/rrwick/Porechop) and filtered with Filtlong (https://github.com/rrwick/Filtlong) using a Phred score of 30. In addition, we used publicly available paired-end Illumina sequence data for each strain (30).

### Genome assembly, annotation, and SV detection

Genome assembly was performed with Fly v2.9 (--nano-hq -g 400 m) (60). In addition, three rounds of nanopolish (https://github.com/jts/nanopolish) and pilon (https://github.com/broad institute/pilon) were carried out. The raw assembly was polished using Illumina reads filtered with a Phred score of 30 (Burrows-Wheeler Aligner). The genome assembly was annotated using the LRSDAY pipeline (61) and the *L. cidri* CBS2950 reference genome as a model. To identify the SVs in *L. cidri* strains, we performed pairwise comparisons between the *de novo* long-read assemblies of the nine strains using MUM&Co v3.8 (62). The pipeline used MUMmer v4 (63) to perform whole-genome alignments and detect SVs ≥ 50 bp.

### Phylogenetic analysis

A maximum-likelihood phylogenetic tree was constructed using protein sequences predicted from *L. cidri* strains and published genomes of *the Lachancea* genus strains available in GRYC: *Lachancea mirantina*, *Lachancea kluyveri,* and *Lachancea fermentati*. Ortholog-Finder v2.4.1 (64) was employed to identify orthologous protein groups among these *Lachancea* species. Subsequently, a total of 3,408 single-copy orthologs identified in all species were aligned with Muscle v3.8.15, after which poorly aligned positions were trimmed with Gblocks v0.91v. 3,408 alignments were concatenated to produce a maximum-likelihood tree with RAxML v8.2.12 (-f a -x 12,345 p 12345 -# 100 m PROTGAMMAJTT -k).

### RNA sequencing and differential expression analysis

Gene expression analysis was performed on strains *L. cidri* CBS2950 and *L. cidri* LC1, which exhibited a significant difference in fermentative kinetics under low nitrogen conditions (SWM60). The two strains were fermented in SWM60 in triplicates and RNA was obtained as previously described (58). RNA integrity was confirmed using Fragment Analyzer (Agilent). RNA-seq libraries and reads trimming were performed as previously described in reference (58). Cleaned reads were mapped to the *L. cidri* CBS2950 genome assembly using HISAT2 v2.2.1 (--max-intronlen 25000 --dta-cufflinks) (65). DEG analysis was performed using the DESeq2 package in R v4.1.2, comparing the two strains (66). Genes with an FDR < 0.05 and an absolute value of fold change >1.5 were considered DEGs for the comparison, *L. cidri* CBS2950 vs *L. cidri* LC1. Gene Ontology analysis was performed with the R package enrichGO.

### Growth under proline as nitrogen source

Cells were pre-cultivated at 20°C without agitation for 48 hours in 96-well plates containing 200 µL YNB (Yeast Nitrogen Base w/o ammonium and amino acids; Difco) medium supplemented with 2% (wt/vol) glucose. A volume of 10 µL of pre-inoculum was used to inoculate a new 96-well plate containing 200 µL of YNB w/o ammonium and amino acids with different concentrations of proline (200, 100, and 50 mg mL YAN) to an optical density (OD_600_) of 0.03–0.1. OD_600_ for each well was measured at 620 nm every 30 minutes for 250 hours. As a control, we used a combination of four different amino acids (glutamic acid, aspartic acid, alanine, and leucine) at 200 and 50 mg/mL YAN. From these data, three parameters were estimated: lag phase, growth rate (μmax), and maximum OD using the GrowthRates software (67).

### Ethanol and sulfite tolerance screening

Ethanol tolerance was evaluated in agar plates (YNB-agar 2%) supplemented with different ethanol concentrations (6, 8, 12, and 15% vol/vol). Yeast cells were cultivated to exponential phase, then spotted on 10-fold serial dilutions on agar plates and incubated at 20°C for 3 days. Sulfite tolerance assays were performed as described previously (24). Microplates were sealed with Breathe-Easy® sealing membranes (Diversified Biotech, USA) and incubated at 28°C. OD_600_ was measured after five days using a Tecan Infinite M200 plate reader (Tecan, Austria).

### Statistical analyses

All phenotype experiments were carried out at least in triplicate. All statistical analyses (*t*-test, one-way ANOVA with correction for multiple comparisons) were performed in R software (68). A *P*-value < 0.05 was considered statistically significant.

## Data Availability

All fastq sequences were deposited in the National Center for Biotechnology Information (NCBI) as a Sequence Read Archive under the BioProject accession number PRJNA978517.

## SUPPLEMENTAL MATERIAL

Supplemental Figures msystems.01058-23-s0001.pdf Figures S1 to S8.

Supplemental Tables msystems.01058-23-s0002.xlsx Tables S1 to S10.

ASM does not own the copyrights to Supplemental Material that may be linked to, or accessed through, an article. The authors have granted ASM a non-exclusive, world-wide license to publish the Supplemental Material files. Please contact the corresponding author directly for reuse.
