# Supplementary material for: Domestication signatures in the non-conventional yeast Lachancea cidri
Source: mSystems. 2023 Dec 12;9(1):e01058-23. doi: 10.1128/msystems.01058-23 (PMC10805023; doi:10.1128/msystems.01058-23)
Supplement: Supplemental Figures — Figures S1 to S8. [file msystems.01058-23-s0001.pdf]

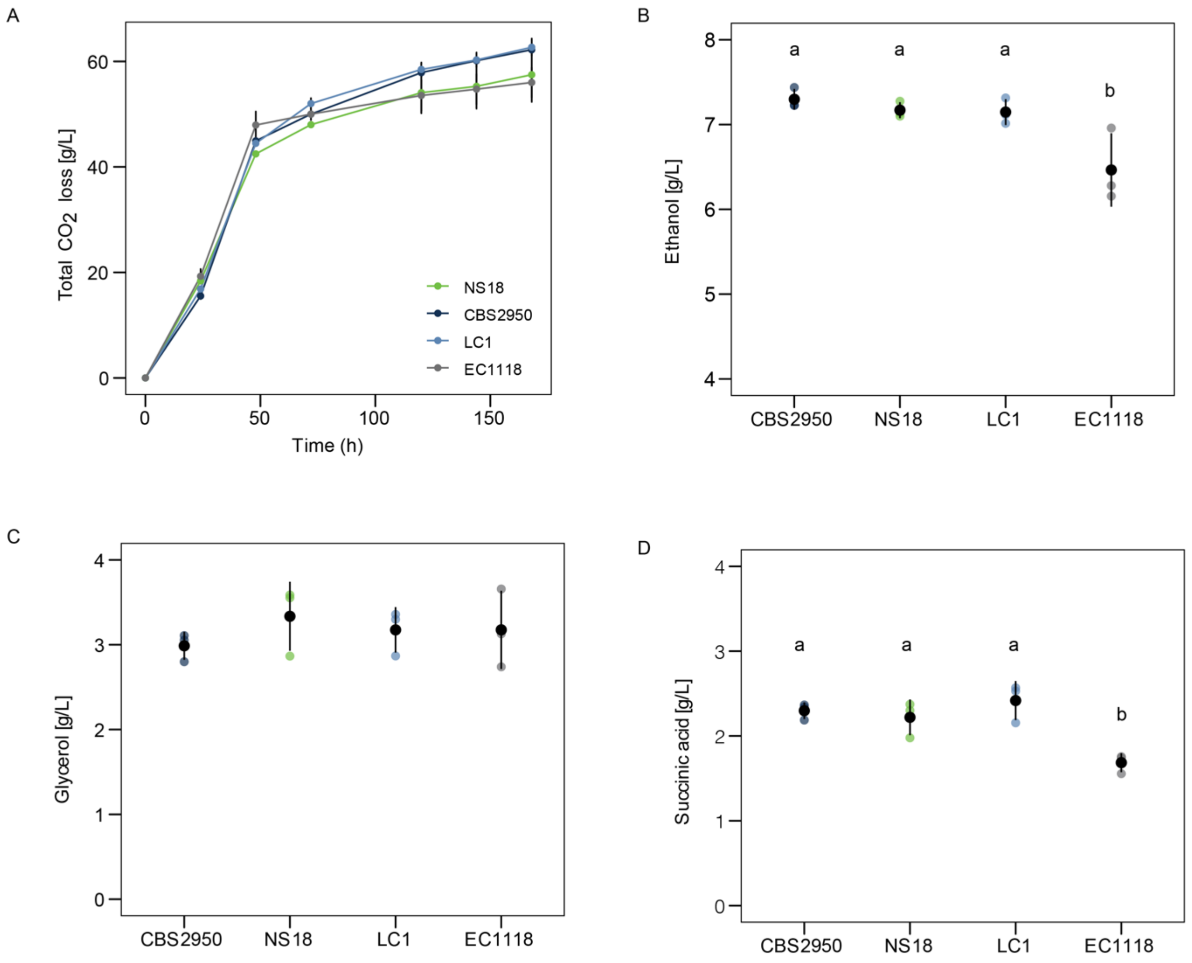

Figure S1. *Lachancea cidri* fermentation performance in Cider. (A) Fermentation kinetics of wild and human-related strains. (B) Ethanol production [g/L]. (C) Glycerol production [g/L]. (D) Succinic acid production [g/L]. Different letters reflect statistically differences between strains with a p-value < 0.05, one-way analysis of variance (ANOVA).

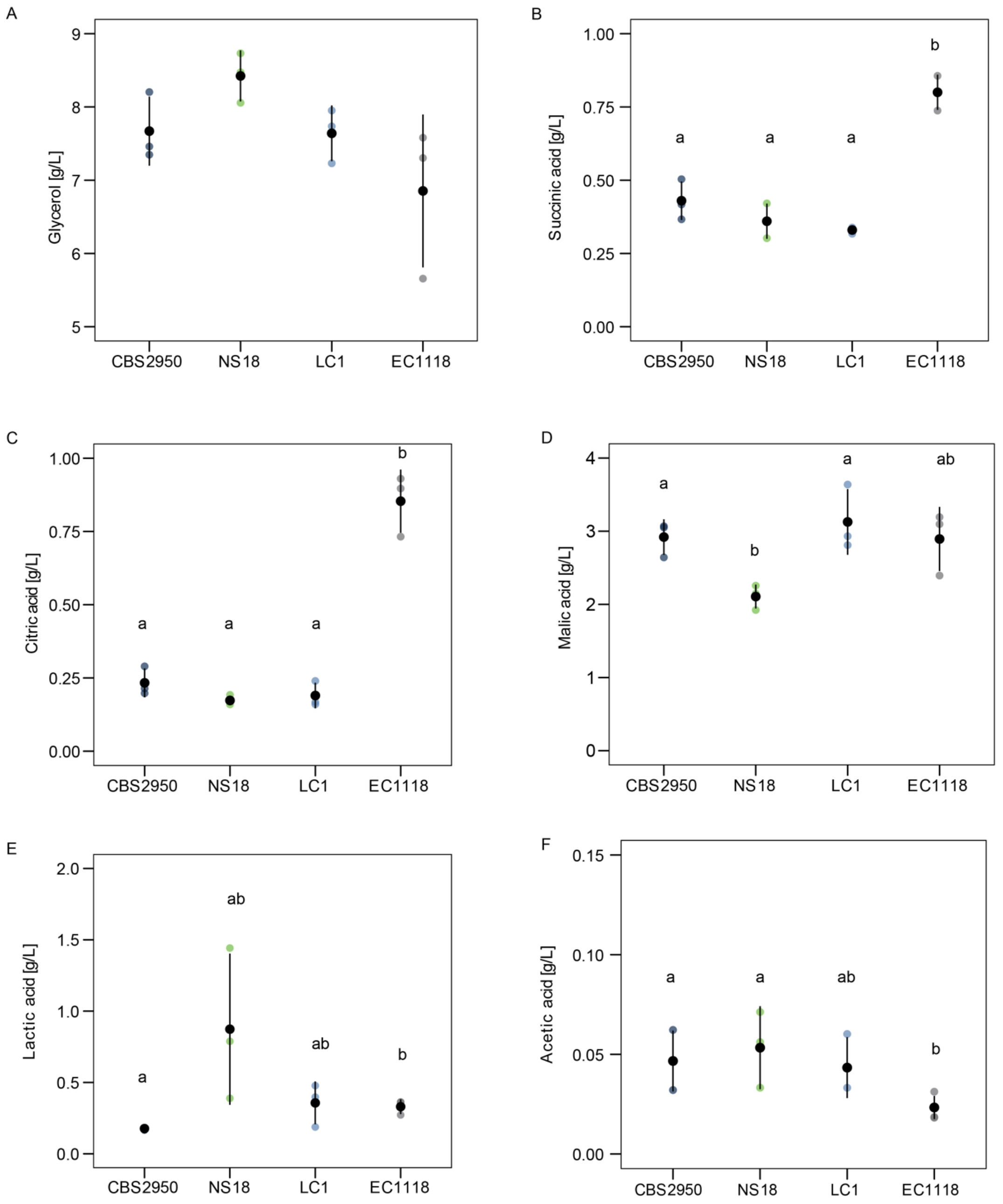

Figure S2. Metabolites produced at the end of Synthetic Wine Must fermentation. (A). Glycerol production [g/L]. (B) Succinic acid production [g/L] (C) Citric acid production [g/L]. (D) Malic acid production [g/L]. (E) Lactic acid production [g/L]. (F) Acetic acid production [g/L]. Different letters reflect statistically differences between strains with a *p*-value < 0.05, one-way analysis of variance (ANOVA).

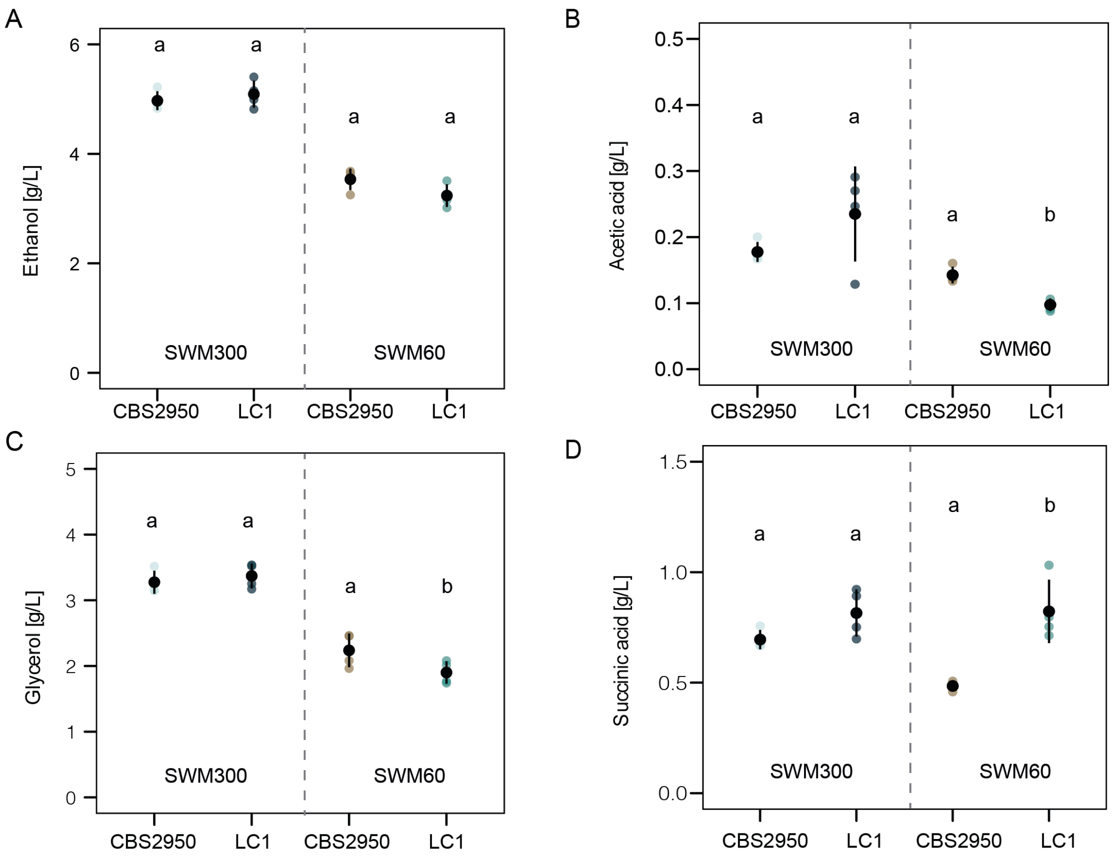

Figure S3. Metabolites produced at the end of Synthetic Wine Must fermentation. (A). Ethanol [g/L]. (B). Acetic acid [g/L] (C). Glycerol [g/L]. (D) Succinic acid [g/L]. Different letters reflect statistically differences between strains with a *p*-value < 0.05, one-way analysis of variance (ANOVA).

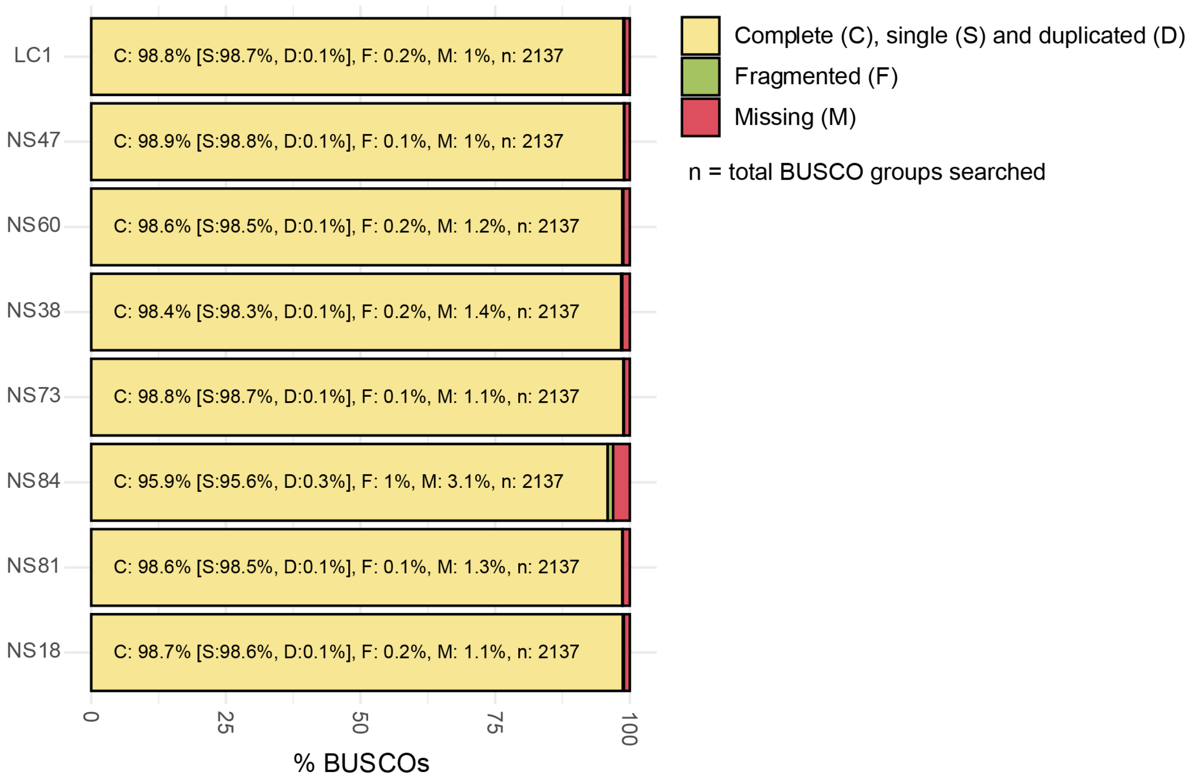

Figure S4. BUSCO's completeness of each assembly.

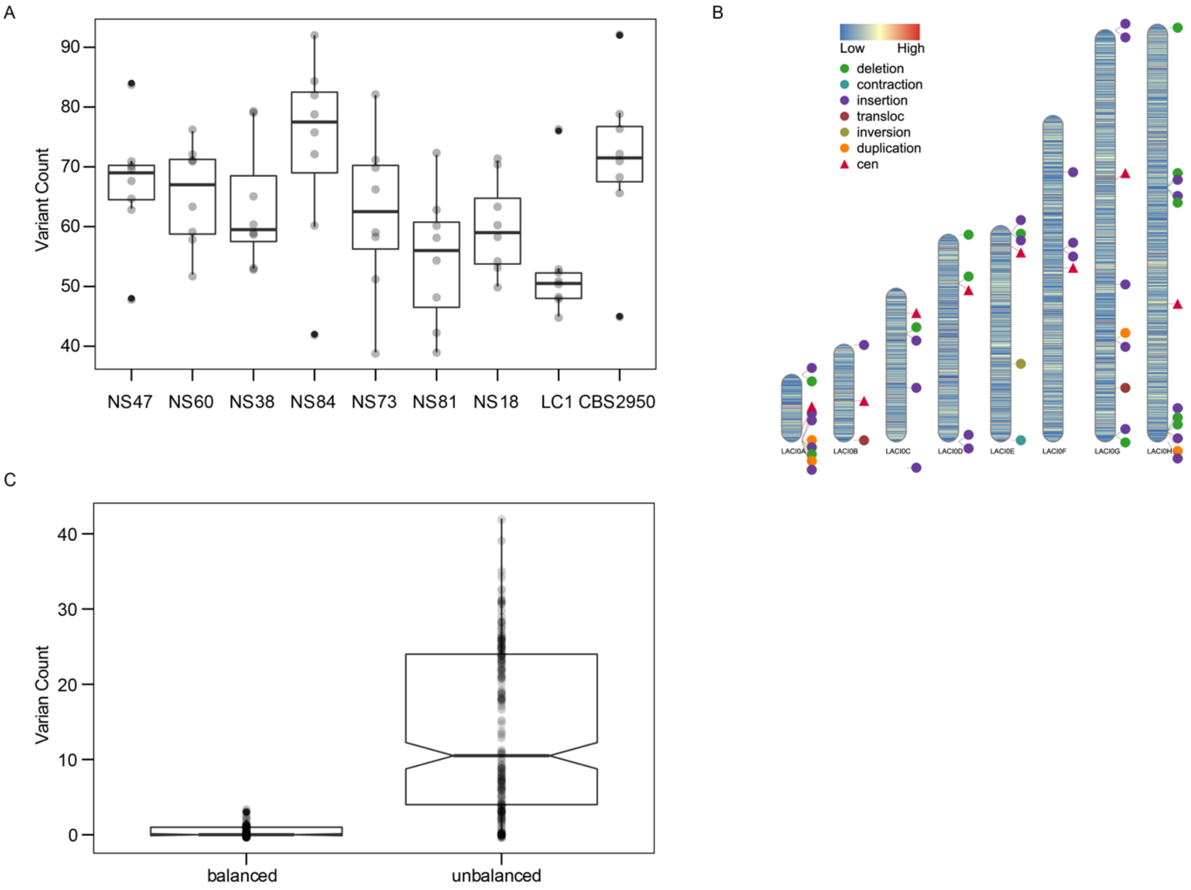

Figure S5. Structural variations within *Lachancea cidri* strains. (A). The range of total structural variation counts found for each genome serves as the reference genome. (B). SV distribution in the genome between LC1 and CBS2950 (C). Balanced and Unbalanced variant count.

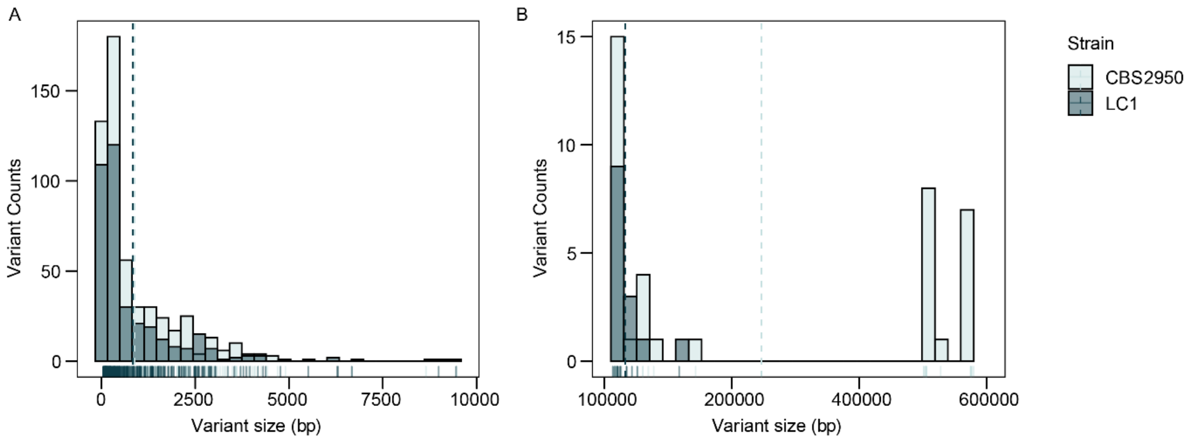

Figure S6. Distribution of SV sizes between *L. cidri* CBS2950 and LC1. (A). SV < 10,000 bp. (B). SV > 10,000 bp.

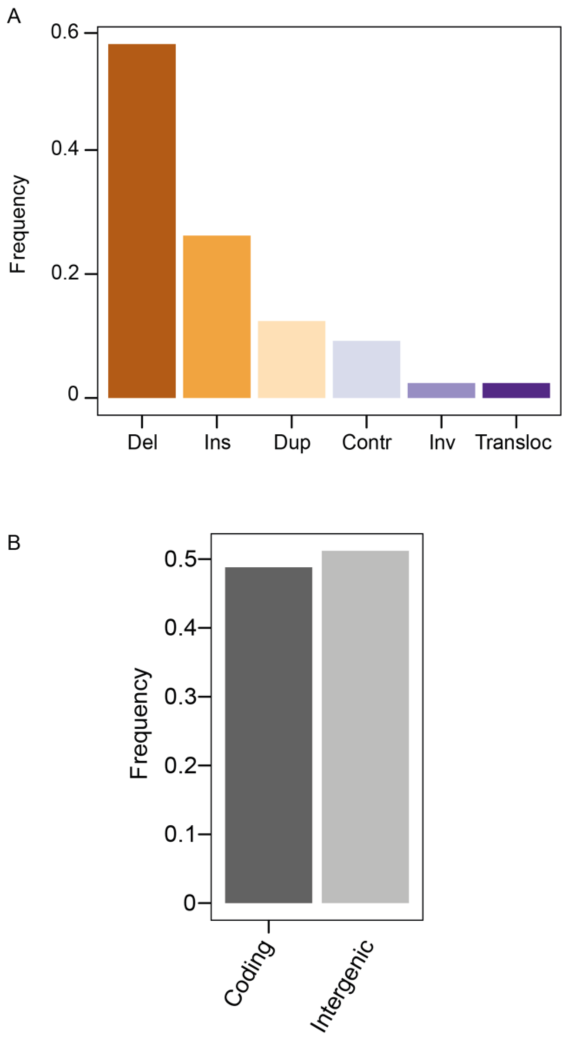

Figure S7. (A) Frequency of SVs between CBS2950 and LC1 (Del for deletions, Ins for insertions, Dup for duplications, Inv for inversions, Contr for contractions, and Transloc for translocations) (B) Frequency of SVs located in coding or non-coding regions between CBS2950 and LC1 strains.

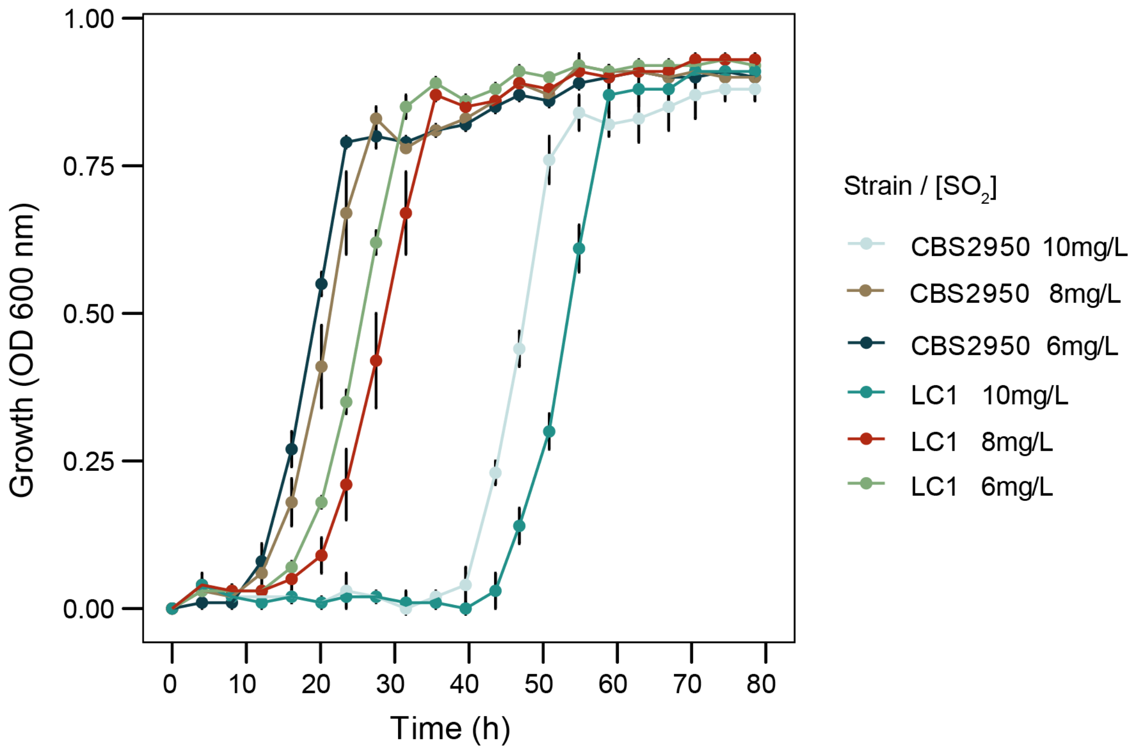

Figure S8. Growth kinetics at different sulfite concentrations.
